# Are Husbands Involving in Their Spouses’ Utilization of Maternal Care Services?: A Cross-Sectional Study in Yangon, Myanmar

**DOI:** 10.1371/journal.pone.0144135

**Published:** 2015-12-07

**Authors:** Kyi Mar Wai, Akira Shibanuma, Nwe Nwe Oo, Toki Jennifer Fillman, Yu Mon Saw, Masamine Jimba

**Affiliations:** 1 Department of Human Ecology, Graduate School of Medicine, the University of Tokyo, Tokyo, Japan; 2 Department of Community and Global Health, Graduate School of Medicine, the University of Tokyo, Tokyo, Japan; 3 Department of Medical Research (Lower) Myanmar, Yangon, Myanmar; 4 Women Leaders Program to Promote Well-being in Asia, the Graduate School of Medicine, Nagoya University, Nagoya, Japan; National Institute of Health, ITALY

## Abstract

**Introduction:**

Husbands can play a crucial role in pregnancy and childbirth, especially in patriarchal societies of developing countries. In Myanmar, despite the critical influence of husbands on the health of mothers and newborns, their roles in maternal health have not been well explored. Therefore, the aim of this study was to identify the factors associated with husbands’ involvement in maternal health in Myanmar. This study also examined the associations between husbands’ involvement and their spouses’ utilization of maternal care services during antenatal, delivery and postnatal periods.

**Methods:**

A community-based, cross sectional study was conducted with 426 husbands in Thingangyun Township, Yangon, Myanmar. Participants were husbands aged 18 years or older who had at least one child within two years at the time of interview. Face to face interviews were conducted using a pretested structured questionnaire. Factors associated with the characteristics of husband’s involvement as well as their spouses’ utilization of maternal care services were analyzed by multivariable logistic regression models.

**Results:**

Of 426 husbands, 64.8% accompanied their spouses for an antenatal visit more than once while 51.6% accompanied them for a postnatal visit. Husbands were major financial supporters for both antenatal (95.8%) and postnatal care (68.5%). Overall, 69.7% were involved in decision making about the place of delivery. Regarding birth preparedness, the majority of husbands prepared for skilled birth attendance (91.1%), delivery place (83.6%), and money saving (81.7%) before their spouses gave birth. In contrast, fewer planned for a potential blood donor (15.5%) and a safe delivery kit (21.1%). In the context of maternal health, predictors of husband’s involvement were parity, educational level, type of marriage, decision making level in family, exposure to maternal health education and perception of risk during pregnancy and childbirth. Increased utilization of maternal health services was found among spouses of husbands who accompanied them to antenatal visits (AOR 5.82, 95% CI, 3.34–10.15) and those who had a well birth plan (AOR 2.42, 95% CI, 1.34–4.39 for antenatal visit and AOR 2.88, 95% CI, 1.52–5.47 for postnatal visit).

**Conclusion:**

The majority of husbands supported their spouses’ maternal care services use financially; however, they were less involved in birth preparedness and postnatal care. Exposure to maternal health education and their maternal health knowledge were main predictors of their involvement. Women were more likely to use maternal care services when their husbands company them for ANC visits and had a well-birth plan in advance.

## Introduction

Pregnancy and childbirth could threaten a woman’s life because of obstetric complications. Annually, worldwide maternal deaths contribute to more than half a million of deaths, and of these, 99% occur in developing countries [1, 2]. According to the World Health Organization (WHO), developing countries accounted for 286,000 of maternal deaths as a result of preventable complications in 2013 [2, 3]. Since appropriate health care services could diminish maternal morbidity and mortality, quality maternal care services should be utilized [4–7].

Husbands’ role is critical in pregnancy and childbirth of women, especially in making a decision about seeking appropriate health care services [8–12]. In patriarchal societies, pregnancy and child birth were often regarded as the women’s exclusive concern [9, 13, 14]. However, husbands are potentially involved in three levels of delays in obstetric emergencies: namely delay in awareness of emergency, delay in seeking care and delay in access to health care providers [15–18]. They are also becoming recognized as key players as their unsupportive attitude could cause a negative impact on the health of their spouses and children [15, 19, 20]. Therefore, since 2001, WHO incorporated husbands into reproductive health programs to achieve safe motherhood [20, 21].

Husbands’ involvement has so far improved the use of reproductive health services, birth preparedness and psycho-social well-being for their spouses [20, 22–24]. For example, husband participation in voluntary HIV counseling and testing was positively associated with uptake of HIV transmission prevention interventions in Kenya [23]. Similarly, in India, husband antenatal attendance was associated with increased skilled birth attendance [25]. Moreover, in Nepal, by educating both husbands and wives in couples, more wives used maternal health services, compared to educating wives alone [22].

Myanmar is still progressing towards meeting the fifth Millennium Development Goal (MDG). The maternal death rate is estimated at over 300 deaths per 100,000 live births [26–29]. Myanmar lags behind most Asian countries such as Vietnam, India and Malaysia in achieving MDG 5 [26–29]. Although the government invests more to improve maternal health services, Myanmar women continue to use these services unsatisfactorily, reflecting the insufficient progress towards MDG 5 [29–32]. In Myanmar, economic, socio-cultural and geographical factors solely or jointly determine a woman’s decision to uptake maternal health services [33, 34]. Meanwhile, husbands’ participation in maternal health also predisposes a challenge on the women’s uptake of maternal health care services in Myanmar [35].

The determinants of husbands’ involvement in maternal care have been explored. However, most of them were either qualitative or descriptive studies [8, 9, 13, 36] or included only limited characteristics of husbands’ involvement [21, 36–38]. In addition, only a few studies have examined the characteristics of husbands’ involvement in three different phases including antenatal, delivery, and postnatal periods [8, 9, 39]. Although husbands critically influence maternal health, little has been explored about the role of husbands in maternal health. Moreover, little is known about how husbands are involved in their spouses’ utilization of maternal care services in Myanmar. Therefore, this study aimed to identify the level of husbands’ involvement in maternal care services and its associated factors in Myanmar. This study also identified the associations between husbands’ involvement and their spouses’ utilization of maternal care services.

## Methods

### Study design and setting

A cross-sectional study was conducted in Thingangyun Township, Yangon, Myanmar in June and July 2014. Myanmar has 15 regions; a region is composed of districts, and each district is then subdivided into townships. A township is composed of village tracts in rural areas and towns or wards in urban areas [40]. Thingagyun Township consists of 38 wards, covering an estimated 14.8 square kilometer with a total population of 194,173 (5,925 children were under two years of age) in 2014.

Eligible participants were husbands aged 18 years and above who had at least one child within two years at the time of interview. A total of 433 eligible participants were recruited. For sampling, a two-staged random sampling method was adopted. In the first stage, 20 wards were randomly selected from total 38 wards. In the second stage, out of assumed 5,925 husbands, 433 eligible participants were selected from the list of fathers of children under the extended program on immunization (EPI) obtained from the township health center. This township health center manages the EPI program of all wards, including the outreach programs.

### Data collection

The tool for data collection was a structured questionnaire (S1 Appendix). The questionnaire items were adapted from survey tools and indicators for maternal and newborn health developed by Jhpiego, an affiliate of Johns Hopkins University [41], Demographic Health Survey (DHS) and relevant prior studies [8, 9, 34]. It comprised four parts: covering 1) socio-demographic characteristics of participants; 2) knowledge of risk of pregnancy and childbirth; 3) assessment of husbands’ involvement in maternal care; and 4) cue for involvement in utilization of maternal care services. The questionnaire was initially prepared in English and translated into the Myanmar Language. To enhance the accuracy of the translation, it was back translated into English by two independent local medical doctors who were familiar with maternal health care. Face to face interviews were conducted by the lead researcher and four trained research assistants. Each interview took about 30 minutes on average. Before collecting the data, the lead researcher conducted a training program for the research assistants to enable them to be familiar with the concept of this study and understand the questions. The same questionnaire was also pretested among 20 participants in Mingalar Taungnyunt Township to correct inconsistencies and inappropriate questions.

### Measurements

In this study, husbands’ involvement is the variable of interest. It refers to 1) antenatal care (ANC) accompaniment once or more; 2) financial support for ANC; 3) birth preparedness [saving money for delivery, purchasing a safe delivery kit, planning for a place and skilled birth attendant for delivery, arranging of transportation for delivery, planning for a potential blood donor], 4) involvement in decision making of the delivery place, 5) accompaniment to the place of delivery and 6) PNC accompaniment; and 7) financial support for PNC. The current study examined separately each variable of husband’s involvement and its associated factors.

Dichotomous questions were used to assess the antenatal, delivery and postnatal accompaniment, financial support for ANC and PNC, and the characteristics of birth preparedness. Birth preparedness was measured by scoring one point for each characteristic, and it was categorized as “well planned” or “not well planned”. The cut-off score was three in accordance with the mean score. Moreover, prior studies of birth preparedness applied this approach of categorization [18, 42, 43].

To assess the husband’s involvement in decision making about delivery place, participants were asked “Who made the final decision of where to give birth?” The responses included “yourself”, “your spouse,” “jointly with your spouse,” “other family member,” “health professional,” “friend,” and “others” [18, 41]. Responses were subdivided into two categories of husbands involved in decision making (“yourself” and “jointly with your spouse”) and others involved.

Independent variables included 1) socio-demographic characteristics including age, ethnicity, religion, education, occupation, monthly income, type of marriage, parity and spouse’s characteristics [8, 9, 21, 36]; 2) knowledge of the risk of pregnancy and childbirth accessed by their knowledge of the danger signs of pregnancy and childbirth. It was measured by using a single question with multiple danger signs. One point was scored for each danger sign and the total score was summed [41]; 3) perception of pregnancy and childbirth was accessed by whether or not they perceived pregnancy and childbirth as life-threatening conditions and maternal care as a necessity [9]; 4) cue for involvement in maternal care refers to any facilitation for husbands to be involved in maternal health. It was assessed by whether participants had ever received any maternal health education and their past experiences about pregnancy and childbirth [21, 41].

Dependent variables were the utilization of maternal care services. These included four or more antenatal visits, choice of delivery care services as home delivery (with skilled or unskilled birth attendant) or institutional delivery (public or private), and receiving PNC [21, 37]. Dichotomous questions were asked to measure the utilization of maternal care services.

### Data analysis

Statistical Analysis was done by Stata 13.1. Descriptive analysis was used to summarize the socio-demographic characteristics and the level of husband’s involvement. Different multiple logistic regression models were constructed to access the factors associated with each of the characteristics of husband’s involvement. Later, three different multiple logistic regression models were run to identify the associations between husbands’ involvement and their spouses’ utilization of maternal care services. The models included the variables which have theoretical or rational associations in prior studies [8, 9, 21, 36, 37]. The significance level was set at a p-value of <0.05.

### Ethical considerations

This study was approved by the Research Ethics Committee of the Graduate School of Medicine, the University of Tokyo, Japan. Ethical approval was also obtained from the Department of Medical Research (Lower Myanmar), Yangon, Myanmar. The study objectives were explained to the participants before each interview. Confidentiality was assured and written informed consent was taken from all participants.

## Results

### Background characteristics of participants

Of 433 husbands, 426 husbands completed the interview in this study (response rate = 98.4%). Table 1 shows the socio-demographic characteristics of husbands. Their mean age was 34.0 (Standard deviation [SD] 6.9) years while that of their spouses was 30.7 (SD 6.5) years. Of 426 husbands, 75.6% followed Buddhism and 68.3% were Bamar. More than half completed high school or higher levels of education (68.1%). The majority of them acquired monogamous marriage (95.3%). Husbands mainly decided for the health care in families (58.4%), followed by jointly with spouse (22.2%), spouse alone (13.2%) and the others (5.6%).

**Table 1:** Background Characteristics of Participants (n = 426).

Characteristics		n	%	Mean	SD
Age (years)		426		34.0	6.9
Religion					
	Buddhist	322	75.6		
	Others	104	24.4		
Ethnicity					
	Bamar	291	68.3		
	National Races**†**	67	15.7		
	Others	68	16.0		
Education					
	Middle school or lower	136	31.9		
	High school or higher	290	68.1		
Occupation					
	Government worker	45	10.6		
	Private/company worker	52	12.2		
	Labor or driver	144	33.8		
	Own business	144	33.8		
	Unemployed or others	41	9.6		
Income per month (USD)#		426		236.2	172.9
Income situation					
	Regular	181	42.5		
	Non-regular	242	56.8		
	Missing	3	0.7		
Type of marriage					
	Monogamous	406	95.3		
	Polygamous	20	4.7		
Currently living with spouse					
	No	18	4.2		
	Yes	408	95.8		
Number of children					
	Only one	193	45.3		
	More than one	233	54.7		
Spouse’s age (years)		426		30.7	6.5
Spouse’s education					
	Middle school or lower	164	38.5		
	High school or higher	262	61.5		
Spouse’s occupation					
	Unemployed/house-wife	300	70.4		
	Employed	126	29.6		
Decision maker regarding health care in family					
	Yourself	249	58.5		
	Your spouse	56	13.1		
	Jointly with your spouse	97	22.2		
	Others	24	5.6		

†National races include Kachin, Kayar, Kayin, Chin, Mon, Yakhine and Shan.

# 1USD = 957.9 kyats, as of 1^st^ July, 2014.

### Husband’s involvement in maternal care

**Fig 1:**
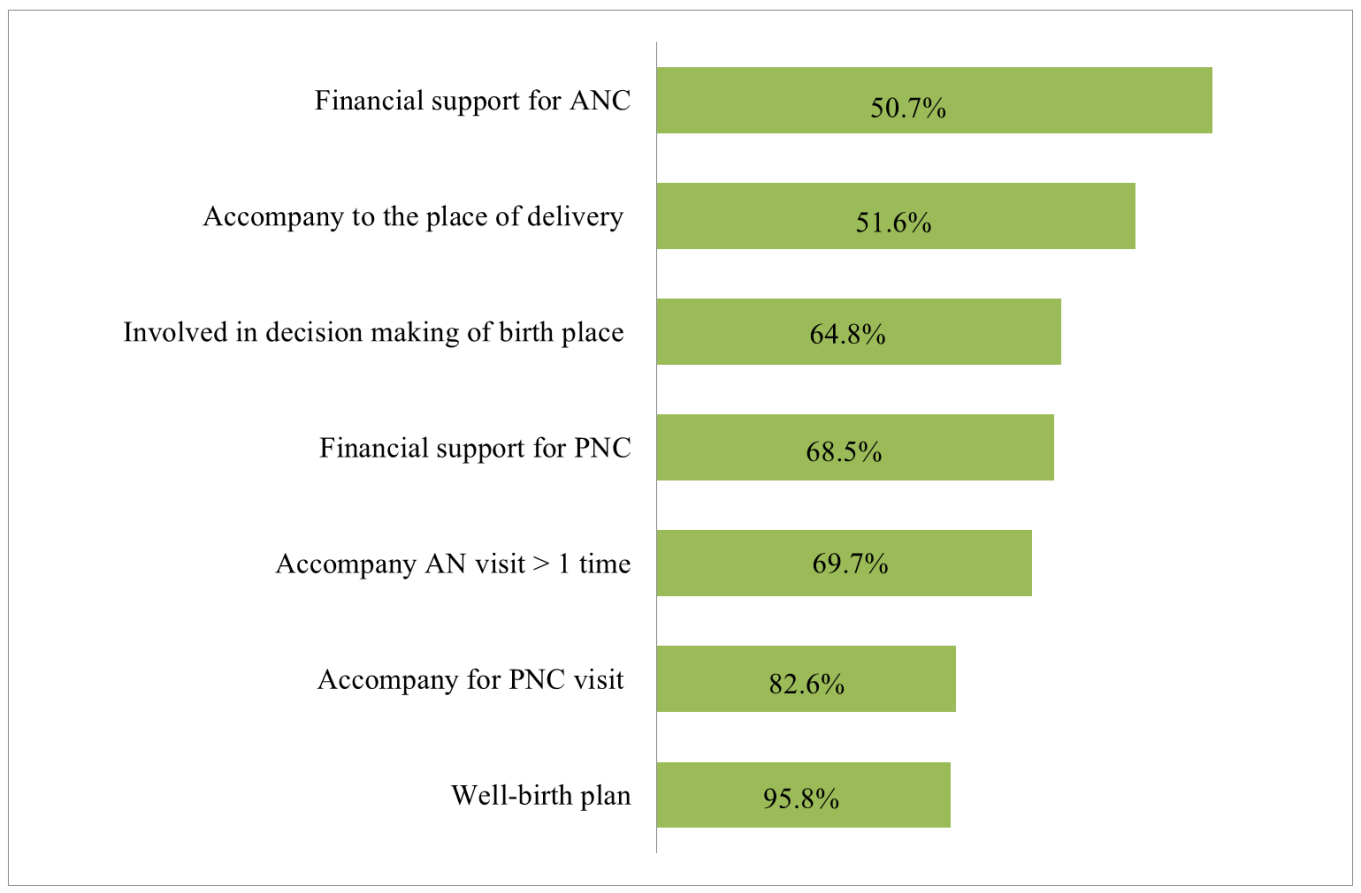
Level of Husband’s Involvement in Maternal Care (n = 426).

As shown in Fig 1, 276 husbands (64.8%) accompanied their spouses’ antenatal visit more than once while 408 (95.8%) provided money for ANC. A total of 279 (69.7%) were involved in decision making about delivery places. Regarding PNC, 51.6% accompanied their spouses and 68.5% provided financial support. Table 2 presents more insight into husbands’ involvement regarding the birth preparedness. Only small percentages of husbands prepared for a potential blood donor (15.5%) and safe delivery kit (21.1%). The majority planned for the delivery place (83.6%), skilled birth attendant (91.1%) and saved money (81.7%) before their spouses gave birth. About half of them (52.1%) planned for the transportation to the delivery place in advance.

**Table 2:** Characteristics of Birth Preparedness (n = 426).

Characteristics		Number	Percentage (%)
Arrange or save money			
	No	78	18.3
	Yes	348	81.7
Plan for transportation			
	No	204	47.9
	Yes	222	52.1
Plan for a delivery place			
	No	70	16.4
	Yes	356	83.6
Arrange a skilled birth attendant			
	No	38	8.9
	Yes	388	91.1
Purchase a safe delivery kit			
	No	336	78.9
	Yes	90	21.1
Plan for a potential blood donor			
	No	360	84.5
	Yes	66	15.5

Table 3 presents the factors associated with husband’s involvement by the multivariable logistic regressions. Having more than one child was negatively associated with antenatal accompaniment (AOR 0.44, 95% CI 0.24–0.81, p = 0.008), having a well-birth plan (AOR 0.44, 95% CI 0.26–0.75, p = 0.003), postnatal accompaniment (AOR 0.24, 95% CI 0.13–0.43, p < 0.001) and financial support for PNC (AOR 0.20, 95% CI 0.09–0.46, p < 0.001). Likewise, polygamous marriage had a negative association with antenatal accompaniment (AOR 0.24, 95% CI 0.07–0.78, p < 0.05), delivery care accompaniment (AOR 0.34, 95% CI 0.12–0.96, p < 0.05) and postnatal care accompaniment (AOR 0.21, 95% CI 0.04–0.98, p < 0.05). Educated husbands were more likely to provide financial support for ANC (AOR 6.08, 95% CI 1.48–25.97, p = 0.012). Husbands were involved more in decision making of their spouses’ delivery place when they were the main decision maker in a family. Additionally, exposure to maternal health education was positively associated with antenatal accompaniment more than once (AOR 2.00, 95% CI 1.10–3.62, p = 0.022), having a well-birth plan (AOR 2.86, 95% CI 1.59–5.14, p < 0.001), and postnatal accompaniment (AOR 6.92, 95% CI 3.31–14.47, p < 0.001). Contrary to other studies, age, monthly income and spouse’s occupation had no significant association with husbands’ involvement in this study [8–10, 36].

**Table 3:** Multivariable Analysis for the Factors Associated with Husband’s Involvement in Maternal Care (n = 426).

Variables		Antenatal		Delivery			Postnatal	
		AOR^A^ (95% CI)		AOR^B^ (95% CI)			AOR^C^ (95% CI)	
		Accompany ANC > one time	Financial support for ANC	Well birth-planned	Accompany to the place of delivery	Decision making of a delivery place	Accompany for PNC visit	Financial support for PNC
Age (years)								
	≤ 30 (ref)	1.0						
	>30	1.17(0.61–2.24)	1.03(0.19–5.52)	1.09(0.61–1.94)	0.90(0.40–2.01)	0.84(0.44–1.62)	1.16(0.61–2.22)	1.44(0.56–3.64)
Education								
	Middle school of lower (ref)	1.0						
	High school or higher	1.32(0.76–2.28)	6.08(1.48–25.97)*	1.22(0.74–2.01)	1.16(0.63–2.13)	0.83(0.47–1.47)	1.03(0.55–1.93)	1.09(0.53–2.21)
Spouse’s age (years)								
	≤ 30 (ref)	1.0						
	>30	1.37(0.77–2.41)	0.80 (0.20–3.21)	1.76(1.04–2.98)*	0.82(0.43–1.59)	1.47(0.82–2.64)	1.34(0.74–2.43)	0.57(0.27–1.23)
Number of child/children								
	Only one (ref)	1.0						
	More than one	0.44(0.24–0.81)**	0.34(0.54–2.16)	0.44(0.26–0.75)**	0.60(0.28–1.27)	0.96(0.51–1.78)	0.24(0.13–0.43)***	0.20(0.09–0.46)***
Type of marriage								
	Monogamous (ref)	1.0						
	Polygamous	0.24(0.07–0.78)*	0.39(0.06–2.47)	0.92(0.30–2.78)	0.34(0.12–0.96)*	1.44(0.46–4.53)	0.21(0.04–0.98)*	0.22(0.04–1.12)
Decision maker regarding health care in family								
	Yourself (ref)	1.0						
	Your spouse	0.84(0.36–1.95)	0.96(0.10–8.72)	0.98(0.45–2.16)	2.56(0.88–7.64)	0.06(0.02–0.13)***	0.80(0.33–1.92)	2.43(0.69–8.55)
	Jointly with your spouse	0.96(0.49–1.87)	0.95(0.17–5.39)	1.08(0.69–2.16)	1.43(0.61–3.37)	1.58(0.73–3.43)	0.46(0.24–1.03)	0.45(0.18–1.09)
	Others	0.80(0.28–2.25)	0.18(0.03–1.14)	0.70(0.27–1.79)	0.56(0.19–1.66)	0.07(0.02–0.20)***	0.34(0.11–1.03)	0.42(0.11–1.53)
Have you received any information regarding with maternal health?		2.00(1.10–3.62)**	2.05(0.57–7.42)	2.86(1.59–5.13)***	3.17(1.61–6.26)**	2.03(1.07–3.85)*	6.92(3.31–14.47)***	8.39(4.06–17.38)***
	No (ref)	1.0						
	Yes	2.00(1.10–3.62)*	2.05(0.57–7.42)	2.86(1.59–5.13)***	3.17(1.61–6.26)**	2.03(1.07–3.85)**	6.92(3.31–14.47)***	8.39(4.06–17.38)***
Knowledge about danger signs of pregnancy, childbirth and puperium		1.51(1.07–2.13)**	1.16(0.37–3.65)	2.04(1.48–2.82)***	1.36(0.86–2.16)	1.16(0.83–1.61)	1.36(0.99–1.87)	1.57(0.94–2.62)

*p-value<0.05

**p-value<0.01

***p-value<0.001.

ᴬ Adjusted for age, education, occupation, income per month, spouse’s age, spouse’s occupation, number of child/children, type of marriage, currently living with a spouse, decision maker regarding health care in a family, exposure to maternal health education, perception on risk of pregnancy and childbirth, knowledge about danger signs of pregnancy and childbirth, perception on ANC.

ᴮ Adjusted for age, education, occupation, income per month, spouse’s age, spouse’s occupation, number of child/children, type of marriage, currently living with a spouse, decision maker regarding health care in a family, exposure to maternal health education, perception on risk of pregnancy and childbirth, knowledge on danger signs of pregnancy and childbirth.

ᶜ Adjusted for age, education, occupation, income per month, spouse’s age, spouse’s occupation, number of child/children, type of marriage, currently living with a spouse, decision maker regarding health care in a family, exposure to maternal health education, perception on risk of pregnancy and childbirth, knowledge on danger signs of pregnancy and childbirth, perception on ANC, perception on PNC.

**Fig 2:**
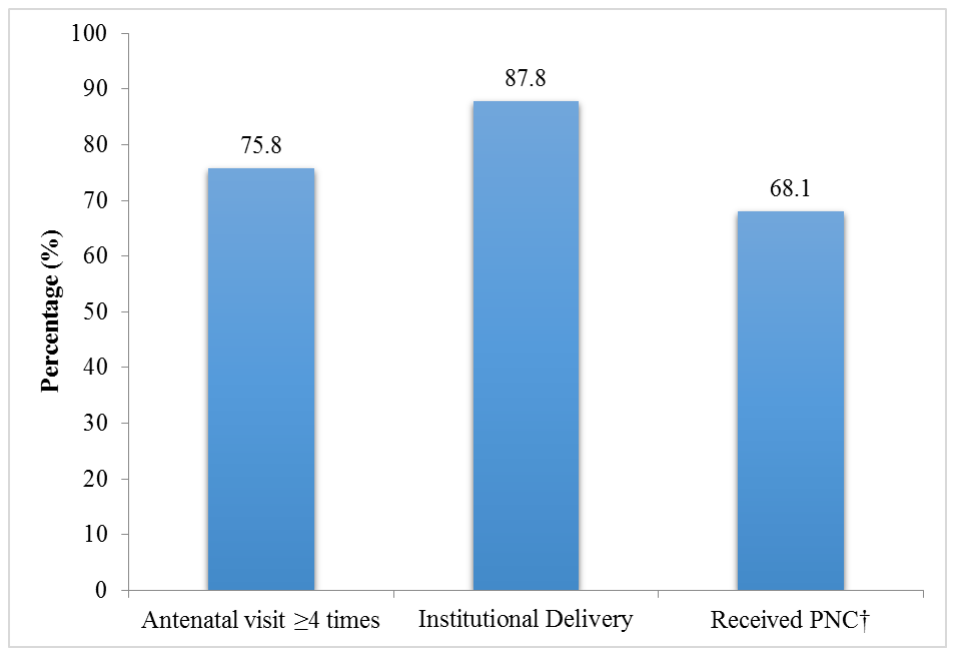
Reported Their Spouses’ Utilization of Maternal Health Care Services (n = 426). †n = 423.

According to Fig 2, of total 426, 75.8% of spouses received ANC more than four times, and the majority of them (87.8%) used institutional delivery care. However, only 68.1% of them sought PNC from health personnel. Table 4 shows the associations between husbands’ involvement and their spouses’ utilization of maternal care services. Based on the bivariate analysis, some characteristics were excluded in multivariable models due to a very small number participant. The excluded characteristics were financial support for ANC, accompaniment to the place of delivery, PNC accompaniment, and financial support for PNC. By analyzing with three different multiple logistic regressions models, it was found that husbands’ ANC accompaniment more than once was positively associated with their spouses’ ANC visits more than four times (AOR 5.82, 95% CI, 3.34–10.15). Similarly, husbands having a well-birth plan had a strong positive association with their spouses’ antenatal visits more than four times (AOR 2.42, 95% CI, 1.34–4.39) and utilization of PNC services (AOR 2.88, 95% CI, 1.52–5.47).

**Table 4:** Multivariable Analysis for the Associations between Husband’s Involvement and Utilization of Maternal Care Services (n = 426).

Variables		Antenatal visit > = 4 times (95%CI)		Institutional delivery (95%CI)		Received PNC†(95%CI)	
		OR	AOR#	OR	AOR#	OR	AOR#
Accompany antenatal visit							
	No and < = one time (ref)	1.0					
	>one time	9.47(5.69–15.77)***	5.82(3.34–10.15)***	4.64(2.50–8.62)***	1.93(0.92–4.04)	4.06(2.63–6.26)***	1.37(0.73–2.61)
Birth preparedness							
	Not well-planned (ref)	1.0					
	Well	4.82(2.90–8.02)***	2.42(1.34–4.39)**	5.92(2.81–12.50)***	2.25(0.96–5.28)	5.94(3.72–9.47)***	2.88(1.52–5.47)**
Decision making of delivery place							
	Others (ref)	1.0					
	Husbands involved	1.77(1.11–2.81)*	1.58(0.90–2.77)	1.25(0.68–2.32)	0.93(0.45–1.93)	1.58(1.02–2.45)*	1.23(0.64–2.53)

*p-value<0.05

**p-value<0.01

***p-value<0.001.

†n = 423.

#adjusted for age, education, occupation, spouse’s education, spouse’s occupation, number of child/children, type of marriage and exposure to maternal health education in addition to above mentioned variables in all three models.

## Discussion

Husband involvement in maternal health was found to be off-balanced in Thingagyun Township. Specifically, their indirect involvement as financial support for maternal care (95.8% for ANC and 68.5% for PNC) was much higher compared to their direct involvement as accompaniment (64.8% for ANC and 51.6% for PNC). The regression models revealed that exposure to maternal health education and their maternal health knowledge were main predictors of their involvement in maternal care. This study also identified that women received maternal health care services more frequently when their husbands accompanied them to an antenatal visit and made a well-birth plan in advance.

As shown in Fig 1, husbands’ ANC and PNC accompaniment was relatively low, compared to their ANC and PNC financial support. The possible explanation could be due to gender norms and social discomfort in being present in a female-dominated environment [13, 36, 44]. Additionally, compared to antenatal and delivery periods, husbands involved less during the postnatal period in this study. PNC services utilization itself is comparatively lower than other periods in many developing countries including Myanmar [45–49]. Moreover, husbands may perceive that wives and children should be taken care by the female family members in the postnatal period, not by the male family members [50, 51]. They may not be aware the services available in the postnatal period or they may not perceive benefits from PNC visits [46]. Therefore, proper PNC should be encouraged with adequate consideration of socio-cultural norms.

This study showed that husbands were involved more in maternal health programs if they had higher maternal health knowledge. With respect to previous studies, lower knowledge on maternal health precluded husbands from positive participation and interest in maternal issues [19, 38, 52]. As a result, those husbands subsided their support on their spouses’ utilization of maternal care services [19, 51]. Therefore, this study highlights a call to recognize husbands as clients of maternal care services.

In this study, having more than one child and polygamous marriage were negatively associated with husband’s involvement. Husbands tend to pay more care and attention to their spouses’ maternal issues when the newborn is their first child or when they are in a monogamous marriage [9, 39]. In a polygamous society, husbands may have difficulty in changing their perception as they do not need to be involved in maternal issues [50]. Moreover, both having more than one child and polygamous marriage were known to be associated with lower utilization of maternal care services in many studies [37, 39, 53]. Thus, husbands’ involvement should be enhanced to mitigate barriers against maternal care services utilization.

Regarding the birth preparedness, husband’s involvement was notably low for some characteristics. For example, less husbands’ involvement was found in planning for transportation to the delivery place (52.1%), purchasing a safe delivery kit (21.1%), and arranging for a potential blood donor (15.5%). Husbands tend to expect that health centers are liable to provide necessary delivery materials. Consequently, this could contribute to low birth-preparedness for some characteristics. Birth preparedness packages have been proven to be an effective strategy in obstetric emergencies to reduce maternal mortality [41–43]. However, the characteristics of birth preparedness are not yet familiar among Myanmar husbands, and awareness should be raised not only for women but also for their partners.

In consistence with previous studies [34, 54], reported utilization of ANC services was 75.8% in this study. Institutional deliveries (87.8%) was much higher than the national 2010 UNFPA report in which 76.4% of deliveries took place at home [54]. These differing results could possibly be due to the peri-urban study setting and easy accessibility to health facilities [6, 34, 49]. This study reinforces how geological distance and proximity to health facilities determines the utilization of health care services.

In Table 4, having more than four skilled antenatal visits was positively associated with husbands’ antenatal accompaniment more than once. Furthermore, spouses were more likely to use skilled ANC when their husbands had well-birth plans. Women’s utilization of safe delivery care was considerably influenced by their husbands’ concern about pregnancy and childbirth in physical, psychological, or social context [5–7, 14]. In the prior studies, husbands’ antenatal accompaniment was considered as their direct involvement, and women had better birth outcomes when their husbands accompanied them to antenatal visits [6, 18]. Therefore, to achieve safe motherhood, one of the critical factors is to encourage husbands to be involved in maternal health.

This study had some limitations. First, this study setting was peri-urban; therefore, the findings could not be generalizable in large cities and rural areas. Second, eligible participants were selected from the list of fathers in the immunization program of children. Therefore, it is possible that some of the eligible participants may not have been in this list. Nevertheless, it may contribute only a small fraction since the immunization coverage is more than 95% in Yangon, Myanmar [30]. Third, recall bias could be present although the study included fathers of children less than two years. Lastly, in this study, the underlying reasons for their less involvement were not assessed in detail.

## Conclusion

In Myanmar, although husbands’ involvement was high for financial support, they were less involved in birth preparedness and PNC as compared to ANC. More husbands’ involvement was found among higher educated husbands and husbands of primigravide. Exposure to maternal health education and their maternal health knowledge could increase their involvement. Husbands’ antenatal accompaniment and having a well birth plan enhanced their spouses’ utilization of maternal care services.

To strengthen husband encouragement, maternal health interventions should target husbands as consumers of maternal health services, hear more on postnatal issues, and raise their awareness about birth preparedness. Further qualitative studies are also recommended to address the underlying reasons of their less involvement and design effective interventions to improve their involvement.

## Supporting Information

S1 Appendix Survey Questionnaire. (DOCX)
