# Correction: Are Husbands Involving in Their Spouses' Utilization of Maternal Care Services?: A Cross-Sectional Study in Yangon, Myanmar

**DOI:** 10.1371/journal.pone.0151295

**Published:** 2016-03-17

**Authors:** Kyi Mar Wai, Akira Shibanuma, Nwe Nwe Oo, Toki Jennifer Fillman, Yu Mon Saw, Masamine Jimba

Fig 1 appears incorrectly in the published article. Please see the correct Fig 1 and its legend here.

**Fig 1:**
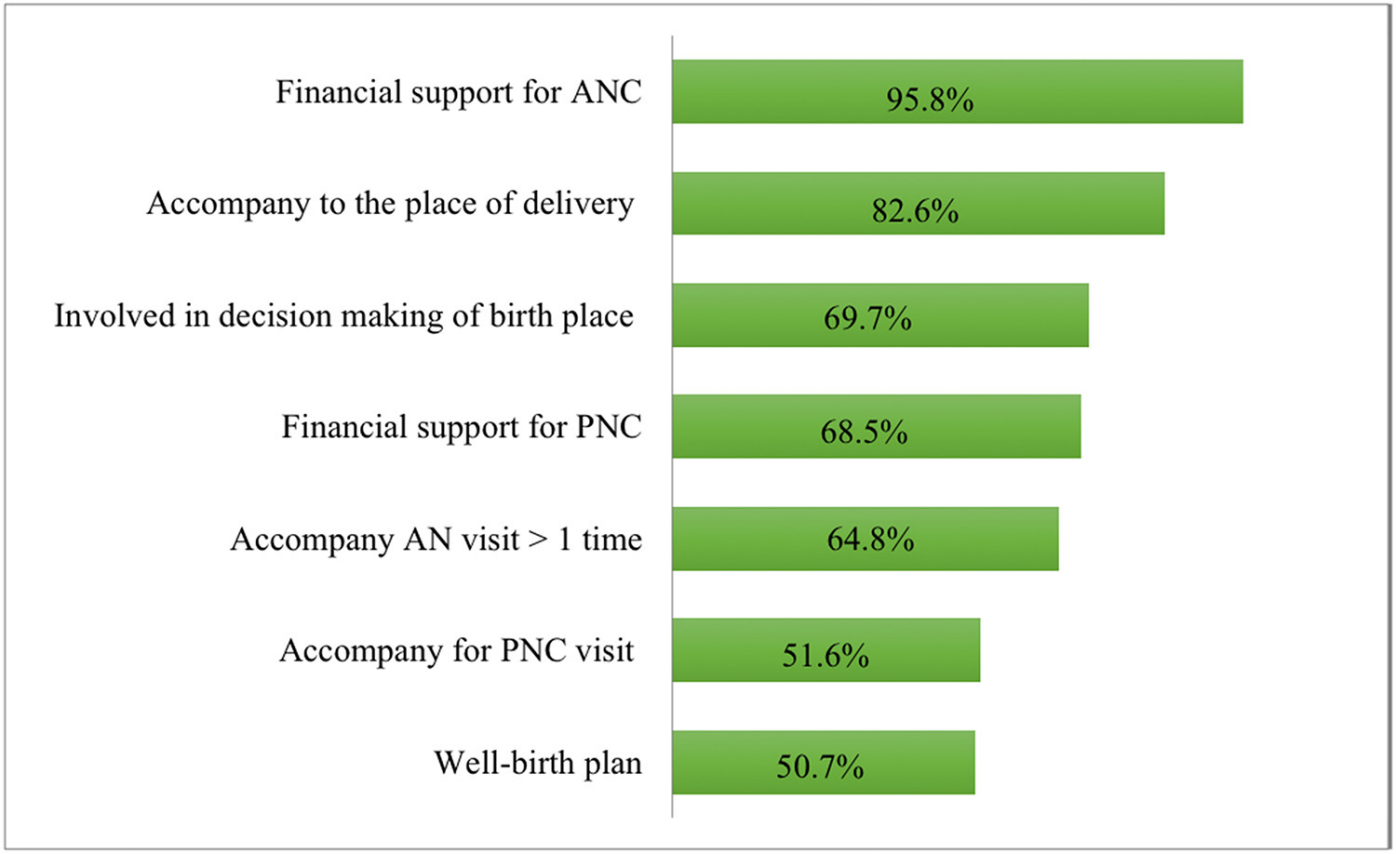
Level of Husband’s Involvement in Maternal Care (n = 426).
