# Supplementary material for: Are Husbands Involving in Their Spouses’ Utilization of Maternal Care Services?: A Cross-Sectional Study in Yangon, Myanmar
Source: PLoS One. 2015 Dec 7;10(12):e0144135. doi: 10.1371/journal.pone.0144135 (PMC4671588; doi:10.1371/journal.pone.0144135)
Supplement: S1 Appendix — (DOCX) [file pone.0144135.s001.docx]

**Questionnaire (English Version)**

**Husbands’ Involvement in their Spouses’ Utilization of Maternal Care Services in Thingangyun Township, Yangon Region, Myanmar**

**SURVEY QUESTIONNAIRE (English version)**

**Identification:**

| **Township:** | **Ward name:** |
| --- | --- |
| **Interviewer**  **Name:** | **Date of interview:** / / |

| **Field Editor** | **Data Entry** |
| --- | --- |
| Name:  Date: / / | Name:  Date: / / |

**Introduction**

Mingalarbar!

My name is . We are interviewing people about men’s involvement in maternity care. The information you give to me is confidential. The result of this survey will be used to help improve health programs for women.

**Eligibility**

Men (aged 18 years or above) who are married and have at least one child within two years, living in Thingangyun Township for more than 6 months.

**Instruction: Circle the answers, which are responded by the participants or fill in the blank areas. Thank you for your participation.**

|  | **Questions** | **Answers** | **Option** | | **Skip** |
| --- | --- | --- | --- | --- | --- |
| **1. Socio-demographic characteristics** | | | | | |
| Q1 | How old are you? (Completed year) |  |  | |  |
| Q2 | What is your religion? | Buddhist | 1 | |  |
|  |  | Christian | 2 | |  |
|  |  | Muslim | 3 | |  |
|  |  | Hindu | 4 | |  |
|  |  | Others | 5 | |  |
| Q3 | What is your ethnicity? | Bamar | 1 | |  |
|  |  | National Races | 2 | |  |
|  |  | Others | 3 | |  |
| Q4 | What is the highest education or year of school you completed? | Illiterate | 1 | |  |
|  |  | Read and write | 2 | |  |
|  |  | Primary School completed | 3 | |  |
|  |  | Middle School completed | 4 | |  |
|  |  | High School completed | 5 | |  |
|  |  | Graduate and above | 6 | |  |
| Q5 | What is your present occupation? | Unemployed | 1 | |  |
|  |  | Government worker | 2 | |  |
|  |  | Private sectors | 3 | |  |
|  |  | Farmer | 4 | |  |
|  |  | Labor or driver | 5 | |  |
|  |  | Own business | 6 | |  |
|  |  | Others | 7 | |  |
| Q6 | How much is your income per month? | In local currency (kyats) | | | Skip if the respondent is unemployed |
| Q7 | What is the situation of your income? | Regular | | 1 | Skip if the respondent is unemployed |
|  |  | Not regular | | 2 |  |
| Q8 | What is the character of your marriage? | Monogamous | | 1 |  |
|  |  | Polygamous | | 2 |  |
| Q9 | Are you currently living with your spouse? | No | | 0 |  |
|  |  | Yes | | 1 |  |
| Q10 | How old is your spouse? |  | |  |  |
| Q11 | What is your spouse’s education? | Illiterate | | 1 |  |
|  |  | Read and write | | 2 |  |
|  |  | Primary school completed | | 3 |  |
|  |  | Middle school completed | | 4 |  |
|  |  | High school completed | | 5 |  |
|  |  | Graduate and above | | 6 |  |
| Q12 | What is your spouse’s occupation? | Unemployed/House wives | | 1 |  |
|  |  | Government worker | | 2 |  |
|  |  | Private sectors | | 3 |  |
|  |  | Farmer | | 4 |  |
|  |  | Labor | | 5 |  |
|  |  | Own business | | 6 |  |
|  |  | Others | | 7 |  |
| Q13 | How much is your spouse’s income per month? | In local currency (kyats) | |  | Skip if your spouse is unemployed |
| Q14 | What is the situation of your spouse’s income? | Regular | | 1 | Skip if your spouse is unemployed |
|  |  | Not regular | | 2 |  |
| Q15 | Who usually makes decisions about health care for the family members? | Yourself | | 1 |  |
|  |  | Your spouse | | 2 |  |
|  |  | Jointly with your spouse | | 3 |  |
|  |  | Others | | 4 |  |
| **2.Knowledge on Risk of Pregnancy and Child Birth** | | | | | |
| Q16 | In your opinion, can unforeseen problems related to pregnancy or childbirth endanger the life of a woman? | No | 0 | |  |
|  |  | Yes | 1 | |  |
|  |  | Don’t know | 2 | |  |
| Q17 | Do you know about the danger signs of pregnancy, childbirth and postnatal period? | No | 0 | |  |
|  |  | Yes | 1 | |  |
| Q18 | If yes, what are they?  Circle what respondents give, then probe (do u think …… is a danger sign?) | Fever | 1 | |  |
|  |  | Bleeding | 2 | |  |
|  |  | Convulsions | 3 | |  |
|  |  | Swollen hands/face | 4 | |  |
|  |  | Severe headache | 5 | |  |
|  |  | Infection | 6 | |  |
|  |  | Others(Specify) | 7 | |  |
| **3. Husband’s involvement in Maternal Care**  **Regarding your spouse’s most recent pregnancy**  **3.1 During Antenatal Period** | | | | | |
| Q19 | Do you think antenatal checkups are necessary? | No | 0 | |  |
|  |  | Yes | 1 | |  |
| Q20 | Did your spouse receive antenatal care (ANC) checkups? | No | 0 | |  |
|  |  | Yes | 1 | |  |
| Q21 | If yes, how many times did she receive? | <4 times | 0 | |  |
|  |  | >= 4 times | 1 | |  |
|  |  | Don’t know | 99 | |  |
| Q22 | In your opinion, who should accompany women for ANC checkups? | Husband | 1 | |  |
|  |  | Mother in law | 2 | |  |
|  |  | Sister in law | 3 | |  |
|  |  | Others | 4 | |  |
| Q23 | Did you accompany your spouse for ANC checkups? | No | 0 | |  |
|  |  | Yes | 1 | |  |
| Q24 | If no, why? | ………………………… |  | |  |
| Q25 | If yes, how many times did you accompany her? | 1 | 0 | |  |
|  |  | One than 1 | 1 | |  |
| Q26 | Did you provide money for transportation/drugs for ANC checkups? | No | 0 | |  |
|  |  | Yes | 1 | |  |
| Q27 | During the antenatal period, did your spouse experience any serious health problems? | No | 0 | |  |
|  |  | Yes | 1 | |  |
| Q28 | If yes, what problems did she experience? | Severe bleeding | 1 | |  |
|  |  | Severe headache | 2 | |  |
|  |  | Convulsion | 3 | |  |
|  |  | High fever | 4 | |  |
|  |  | Loss of consciousness | 5 | |  |
|  |  | Swollen hands/face | 6 | |  |
|  |  | Severe weakness | 7 | |  |
|  |  | Severe abdominal pain | 8 | |  |
|  |  | Water breaks without labor | 9 | |  |
|  |  | Accelerated/reduced fetal movement | 10 | |  |
|  |  | Others(Specify) | 99 | |  |
| **3.2 Birth Preparedness** | | | | | |
| Q29 | When your spouse was pregnant, did you arrange or save the money for delivery? | No | 0 | |  |
|  |  | Yes | 1 | |  |
| Q30 | When your spouse was pregnant, did you plan where she would deliver the child? | No | 0 | |  |
|  |  | Yes | 1 | |  |
| Q31 | When your spouse was pregnant, did you think about or plan what type of transportation your spouse would use to get to the place of delivery? | No | 0 | |  |
|  |  | Yes | 1 | |  |
| Q32 | When your spouse was pregnant, did you plan for a potential blood donor in case for emergency | No | 0 | |  |
|  |  | Yes | 1 | |  |
| Q33 | When your spouse was pregnant, did you purchase a safe delivery kit? | No | 0 | |  |
|  |  | Yes | 1 | |  |
| Q34 | When your spouse was pregnant, did you arrange a skilled birth attendant for delivery? | No | 0 | |  |
|  |  | Yes | 1 | |  |
| Q35 | Where did your spouse deliver? | Home | 1 | | Institution (hospital+ RHC delivery room) |
|  |  | Public hospital/ health center | 2 | |  |
|  |  | Private hospital/health center | 3 | |  |
| Q36 | Did you accompany your spouse to the place where she gave birth? | No | 0 | |  |
|  |  | Yes | 1 | |  |
| Q37 | Who assisted the delivery? | Doctor | 1 | |  |
|  |  | Nurse/Midwife | 2 | |  |
|  |  | TBA/Traditional Healer | 3 | |  |
|  |  | Community Health worker | 4 | |  |
|  |  | Relative/Friend | 5 | |  |
|  |  | Others | 6 | |  |
| Q38 | Who made the final decision where to give birth? | Yourself | 1 | |  |
|  |  | Your spouse | 2 | |  |
|  |  | Jointly with your spouse | 3 | |  |
|  |  | Other family member | 4 | |  |
|  |  | Health professional | 5 | |  |
|  |  | Friend | 6 | |  |
|  |  | Other(specify) | 7 | |  |
| Q39 | How was the child born? | Normal Spontaneous Vaginal Delivery | 1 | |  |
|  |  | Assistant Delivery (vacuum extraction or forceps) | 2 | |  |
|  |  | Cesarean Section | 3 | |  |
| Q40 | During labor and birth, did your spouse experience any serious health problems related to birth? | No | 0 | |  |
|  |  | Yes | 1 | |  |
|  |  | Don’t know | 99 | |  |
| Q41 | If yes, what problems did she experience? | Severe bleeding | 1 | |  |
|  |  | Severe headache | 2 | |  |
|  |  | Convulsion | 3 | |  |
|  |  | High fever | 4 | |  |
|  |  | Loss of consciousness | 5 | |  |
|  |  | Labor lasting >12 hours | 6 | |  |
|  |  | Placenta not delivered 30 minutes after baby | 7 | |  |
|  |  | Others (Specify) | 99 | |  |
| **3.3 During Postnatal Period** | | | | | |
| Q42 | Do you think postnatal checkups are necessary? | No | 0 | |  |
|  |  | Yes | 1 | |  |
| Q43 | Did your spouse seek postnatal care for the last delivery? | No | 0 | |  |
|  |  | Yes | 1 | |  |
|  |  | Don’t know | 99 | |  |
| Q44 | If yes, from whom did she seek? | Doctor | 1 | |  |
|  |  | Nurse/Midwife | 2 | |  |
|  |  | TBA/Traditional Healer | 3 | |  |
|  |  | Community health worker | 4 | |  |
|  |  | Relative/Friend | 5 | |  |
|  |  | Others | 6 | |  |
| Q45 | Did you accompany your spouse for postnatal checkups? | No | 0 | |  |
|  |  | Yes | 1 | |  |
| Q46 | If no, why? | …………………………. |  | |  |
| Q47 | Did you provide money for transportation/drugs? | No | 0 | |  |
|  |  | Yes | 1 | |  |
| **4. Cue to Involve in utilization of Maternal Health Care Services**  **4.1 Regarding maternal health care education** | | | | | |
| Q48 | Have you seen, heard or read any information regarding antenatal, delivery and postnatal care? | No | 0 | |  |
|  |  | Yes | 1 | |  |
| Q49 | If yes, from which source(s) did you get? | Radio | 1 | |  |
|  |  | TV | 2 | |  |
|  |  | Written source | 3 | |  |
|  |  | Health worker | 4 | |  |
|  |  | Others | 5 | |  |
| **4.2 Personal experience related to previous child**  **(**If you have only one child, skip Q 51 to 57) | | | | | |
| Q50 | How many children do you have who are currently living with you? | 1 | 1 | |  |
|  |  | 2 | 2 | |  |
|  |  | 3 | 3 | |  |
|  |  | More than 3 | 4 | |  |
| Q51 | Where was your previous child delivered? | Home | 1 | |  |
|  |  | Public hospital/health center | 2 | |  |
|  |  | Private hospital/health center | 3 | |  |
| Q52 | Who assisted the delivery? | Doctor | 1 | |  |
|  |  | Nurse/Midwife | 2 | |  |
|  |  | TBA/Traditional healer | 3 | |  |
|  |  | Community Health Worker | 4 | |  |
|  |  | Relative/Friend | 5 | |  |
|  |  | Other | 6 | |  |
| Q53 | When your spouse was pregnant, did you plan where she would deliver the child? | No | 0 | |  |
|  |  | Yes | 1 | |  |
| Q54 | Who made the final decision about where to give birth? | Yourself | 1 | |  |
|  |  | Your spouse | 2 | |  |
|  |  | Jointly with your spouse | 3 | |  |
|  |  | Other family member | 4 | |  |
|  |  | Health professional | 5 | |  |
|  |  | Friend | 6 | |  |
|  |  | Other (specify) | 7 | |  |
| Q55 | How was the child born? | Normal Spontaneous Vaginal Delivery | 1 | |  |
|  |  | Assistant Delivery (vacuum extraction or forceps) | 2 | |  |
|  |  | Cesarean Section | 3 | |  |
| Q56 | During labor and birth, did your spouse experience any serious health problems related to birth? | No | 0 | |  |
|  |  | Yes | 1 | |  |
|  |  | Don’t know | 99 | |  |
| Q57 | If yes, what problems did she experience? | Severe bleeding | 1 | |  |
|  |  | Severe headache | 2 | |  |
|  |  | Convulsion | 3 | |  |
|  |  | High fever | 4 | |  |
|  |  | Loss of consciousness | 5 | |  |
|  |  | Labor lasting >12 hours | 6 | |  |
|  |  | Placenta not delivered 30 minutes after delivery | 7 | |  |
|  |  | Other | 99 | |  |
| **4.3 Father’s characteristics** | | | | | |
| Q58 | What is your father’s education? | Illiterate | 1 | |  |
|  |  | Read and write | 2 | |  |
|  |  | Primary School | 3 | |  |
|  |  | Middle School | 4 | |  |
|  |  | High School | 5 | |  |
|  |  | Graduate and above | 6 | |  |
| Q59 | What is your father’s occupation? | Unemployed | 1 | |  |
|  |  | Government worker | 2 | |  |
|  |  | Private sectors | 3 | |  |
|  |  | Farmer | 4 | |  |
|  |  | Labor or driver | 5 | |  |
|  |  | Own business | 6 | |  |
|  |  | Others | 7 | |  |
| Q60 | Did your father provide money and time to the family? | No | 1 | |  |
|  |  | Yes | 2 | |  |
| Q61 | What is the character of your parents’ marriage? | Monogamous | 1 | |  |
|  |  | Polygamous | 2 | |  |

**Thank you for answering the questions!**
